# Phenotyping of Mice with Heart Specific Overexpression of A_2A_-Adenosine Receptors: Evidence for Cardioprotective Effects of A_2A_-Adenosine Receptors

**DOI:** 10.3389/fphar.2018.00013

**Published:** 2018-01-22

**Authors:** Peter Boknik, Katharina Drzewiecki, John Eskandar, Ulrich Gergs, Stephanie Grote-Wessels, Larissa Fabritz, Paulus Kirchhof, Frank U. Müller, Frank Stümpel, Wilhelm Schmitz, Norbert Zimmermann, Uwe Kirchhefer, Joachim Neumann

**Affiliations:** ^1^Institut für Pharmakologie und Toxikologie, Universitätsklinikum Münster, Westfälische Wilhelms-Universität, Münster, Germany; ^2^Institut für Pharmakologie und Toxikologie, Medizinische Fakultät, Martin-Luther-Universität Halle-Wittenberg, Halle, Germany; ^3^Centre for Cardiovascular Sciences, University of Birmingham, Birmingham, United Kingdom; ^4^Bundesinstitut für Arzneimittel und Medizinprodukte, Bonn, Germany

**Keywords:** A_2A_-adenosine receptor, contractility, ischemia, reperfusion, protein phosphorylation

## Abstract

**Background:** Adenosine can be produced in the heart and acts on cardiac adenosine receptors. One of these receptors is the A_2A_-adenosine receptor (A_2A_-AR).

**Methods and Results:** To better understand its role in cardiac function, we generated and characterized mice (A_2A_-TG) which overexpress the human A_2A_-AR in cardiomyocytes. In isolated atrial preparations from A_2A_-TG but not from WT, CGS 21680, an A_2A_-AR agonist, exerted positive inotropic and chronotropic effects. In ventricular preparations from A_2A_-TG but not WT, CGS 21680 increased the cAMP content and the phosphorylation state of phospholamban and of the inhibitory subunit of troponin in A_2A_-TG but not WT. Protein expression of phospholamban, SERCA, triadin, and junctin was unchanged in A_2A_-TG compared to WT. Protein expression of the α-subunit of the stimulatory G-protein was lower in A_2A_-TG than in WT but expression of the α-subunit of the inhibitory G-protein was higher in A_2A_-TG than in WT. While basal hemodynamic parameters like left intraventricular pressure and echocardiographic parameters like the systolic diameter of the interventricular septum were higher in A_2A_-TG than in WT, after β-adrenergic stimulation these differences disappeared. Interestingly, A_2A_-TG hearts sustained global ischemia better than WT.

**Conclusion:** We have successfully generated transgenic mice with cardiospecific overexpression of a functional A_2A_-AR. This receptor is able to increase cardiac function *per se* and after receptor stimulation. It is speculated that this receptor may be useful to sustain contractility in failing human hearts and upon ischemia and reperfusion.

## Introduction

Adenosine elicits multiple effects in the human body. These include effects on cardiac function that have been studied for many years and some effects of adenosine including the downstream signaling mechanisms are similar to those of vagal stimulation. For example, the negative chronotropic effect on the sinus node, the negative dromotropic effect on the AV-node, and the negative inotropic effect in atrial tissue of adenosine (Shryock and Belardinelli, 1997). Extracellular adenosine acts on A_1_-, A_2_-, and A_3_-adenosine receptors (AR). Typically, A_1_-AR inhibits and A_2_-AR stimulates adenylyl cyclase activities (Olsson and Pearson, 1990). The A_2A_-AR was the first of the P_1_-purinoceptor family to be cloned (Libert et al., 1989).

The negative inotropic effects of adenosine in the rat atrium were mediated by inhibition of adenylyl cyclase activity followed by a decreased cAMP content (Linden et al., 1985). But in guinea pig atrium, no adenosine-induced decrease in cAMP content was found (Böhm et al., 1984). Here, the negative inotropic effect of adenosine was inhibited by pertussis toxin (Böhm et al., 1986) and therefore mediated by a pertussis toxin-sensitive G-protein. Probably, an enhanced conductance of potassium in the sarcolemma was involved (Belardinelli and Isenberg, 1983). However, the activation of protein phosphatases has also been suggested (Gupta et al., 1993a,b).

In contrast, in the ventricle of most species, adenosine is nearly ineffective to influence force of contraction but via activation of A_1_-adenosine receptors, it can decrease the effects of β-adrenergic stimulation or in other words the inotropic effects of cAMP-elevating agents are decreased by adenosine [for example forskolin or phosphodiesterase inhibitors; rat (Dobson, 1978, 1983) or guinea pig (Böhm et al., 1984; Brückner et al., 1985)]. Adenosine can also provoke relaxation of the vasculature via activation of A_2A_-AR on smooth muscle cells [human coronaries (Makujina et al., 1992)].

Adenosine is produced in cardiomyocytes and its release there is markedly enhanced during β-adrenergic stimulation, ischemia, or necrosis. In the clinic, adenosine and its precursor ATP can be used to stop supraventricular arrhythmias and therefore, the effects of adenosine in the mammalian heart are of clinical relevance and should be further investigated. It is controversial and may be species dependent (or method dependent) whether A_2A_-AR is functional (increase in cAMP, increase in contractility) in the mammalian heart: some reported a lack of effect [rat (Shryock et al., 1993), guinea-pig (Boknik et al., 1997), rabbit (Kilpatrick et al., 2002)] whereas other reported a functional response [mouse (Morrison et al., 2006), rat (Monahan et al., 2000)].

It is important to note, that A_2A_-receptors in human hearts have been detected on the protein level (Marala and Mustafa, 1998). Moreover, work in isolated perfused A_2A_-KO (constitutive deletion) mouse hearts clearly established that CGS 21680 a classical A_2A_-receptor agonist was selective: only in WT hearts CGS 21680 (up to 1 μM) increased contractility but not in hearts from A_2A_-KO mice (Ledent et al., 1997). However, under basal conditions (no CGS 21680 given) contractility of WT and KO were not different (Ledent et al., 1997; Ashton et al., 2017), suggesting that CGS 21680 is an appropriate tool to assess A_2A_ function in mice. Moreover, there is evidence that A_2A_-receptor stimulation can protect the heart against reperfusion injury [e.g., rat (Cargnoni et al., 1999)].

As part of our long-standing effort to characterize all known adenosine receptors in the mammalian heart, we report here on the generation and phenotyping of a novel transgenic mouse model in which we successfully expressed an inotropically active A_2A_-AR. We used this model to study reperfusion injury in the isolated perfused heart. A progress report of this work has appeared as abstract (Grote-Wessels et al., 2007).

## Materials and Methods

### Isolation of A_2A_-Receptor cDNA and Generation of Transgenic Mice

The investigation conforms to the *Guide for the Care and Use of Laboratory Animals* published by the National Research Council (2011). Animals were handled and maintained to according to approved protocols of the animal welfare committee of the University of Münster, Germany.

The PCR generated human A_2A_-AR cDNA fragment containing a 3′ and 5′ engineered *NotI* digestion site was inserted into a mouse cardiac α-myosin heavy chain promoter expression cassette via *NotI*. The orientation of the cDNA was confirmed by sequencing. The A_2A_-AR cDNA promoter construct was digested with NruI and purified by a cesium chloride gradient centrifugation for injection into the pronuclei of single-cell fertilized mouse embryos. Generation of transgenic (A_2A_-TG) mice was performed by standard procedures (mouse strain: FVB/N). One transgenic line overexpressing the A_2A_-AR under the control of the α-myosin heavy chain (MHC) promoter was established, which was investigated in the present study. Genotypes were identified by PCR analyses of tail-tip DNA using the following primers 5′-acaaagcaggcgatgaag-3′ and 5′-acccttaccccacatagacc-3′. For the reverse transcription 4 μg RNA and random primers were used (Transcriptor High Fidelity cDNA Synthesis Kit, Roche Applied Science, Mannheim, Germany). The PCR reaction was performed using the Ampliqon Taq DNA polymerase (Biomol, Hamburg, Germany) according to the manufacturer’s instructions. For all experiments, 12–30 weeks old A_2A_-TG mice and WT littermates of both sexes were used.

### Western Blot Analysis

Homogenates from ventricular tissue samples were prepared in 300 μl of 10 mM NaHCO_3_ and 100 μl 20% SDS. Crude extracts were incubated at 25°C for 30 min before centrifugation to remove debris and thereafter, the supernatants (= homogenates) were separated and stored at -20°C until further use. Western blot analysis was performed as previously described (Gergs et al., 2004). Briefly, aliquots of 100 μg of protein were loaded per lane and finally, bands were detected using enhanced chemifluorescence (ECF, GE Healthcare, Munich, Germany) together with a Storm^TM^ PhosphorImager (GE Healthcare, Munich, Germany). Following primary antibodies were used in this study: polyclonal rabbit anti calsequestrin, monoclonal mouse anti SERCA2a, polyclonal rabbit anti triadin, and polyclonal rabbit anti junctin (all kindly provided by L.R. Jones, Indianapolis, IN, United States), monoclonal mouse anti PLB (A-1, Badrilla, Leeds, United Kingdom), polyclonal rabbit anti phospho-PLB (antibodies were raised against PLB-peptide phosphorylated at serine-16 or at threonine-17, Badrilla, Leeds, United Kingdom). The characteristics and use of these antibodies has been reported repeatedly by our group (Kirchhefer et al., 2002). The antibodies against TnI and phosphor-TnI were obtained from GE Healthcare (Freiburg, Germany), the antibodies against α-subunits of Gi-protein and Gs-protein were purchased from Calbiochem (Darmstadt, Germany).

### Histological Analysis

Hearts were fixed in buffered 4% formaldehyde and embedded in paraffin. Four micron sections were mounted on polylysine microslides, dewaxed in xylene, rehydrated in sequential decreasing alcohol concentrations and pre-treated in Tris/EDTA-buffer, pH 9 in a pressure cooker for antigen retrieval. After blocking endogenous mouse IgG with Fab Fragment of goat IgG directed against mouse IgG (100 μg mL^-1^, Dianova, Hamburg, Germany) for 1 h at room temperature and washing slides were incubated with mouse primary monoclonal antibody to A_2A_-AR (clone 7F6-G5-A2, 5 μg ml^-1^, Upstate, NY, United States). For detection a goat-anti-mouse antibody conjugated to Alexa Fluor 594 (1:300, Dianova, Hamburg, Germany) was used. Finally, samples were counterstained for approximately 15 s with DAPI and mounted with Vectashield (Vector Laboratories, Burlingame, CA, United States). For imaging a Zeiss Axiophot2 microscope was used, separate color images (blue for DAPI, red for Alexa 594) were merged by AxioVision multichannel image processing (Carl Zeiss Vision GmbH, Germany). Images shown are representative of at least five independent experiments, which gave similar results (Buchwalow et al., 2004).

### Echocardiography

Echocardiography in spontaneously breathing mice was performed under anesthesia with 1.5% isoflurane as described previously (Gergs et al., 2010).

### Left-Ventricular Catheterization

Mice were anesthetized by i.p. injection of avertin (2,2,2-tribromoethanol, Sigma–Aldrich) in 2% solution at a dose of 400 mg kg^-1^ bodyweight and placed on a controlled heating pad (Föhr Medical Instruments, Seeheim-Ober Beerbach, Germany) in supine position. Additional doses of avertin (each 10% of the initial dose) were applied during experiments if appropriate to maintain depth of anesthesia. A miniature pressure-volume catheter (model SPR-839, Millar Instruments, Houston, TX, United States) was inserted via the right carotid artery and placed in the left ventricle. Increasing doses of dobutamine were administered into the cannulated left external jugular vein using an automated syringe pump (B. Braun, Melsungen, Germany). Data were recorded using the MPVS-400 system (Millar Instruments) and Chart5 software (ADInstruments, Bella Vista, NSW, Australia). At the end of experiments, animals were euthanized by avertin overdose, and hearts were excised, weighed, and stored at -80°C until further examination. Hemodynamic data were analyzed using Chart5 software (ADInstruments) and PVAN software (Millar Instruments).

### Isolation of Cardiomyocytes

Ventricular cardiomyocytes were isolated using a published protocol (Kirchhefer et al., 2002). In brief, hearts were perfused for 5 min at 2 ml min^-1^ with a Ca^2+^-free solution (solution A) composed of (in mM) 140 NaCl, 5.8 KCl, 0.5 KH_2_PO_4_, 0.4 NaH_2_PO_4_, 0.9 MgSO_4_, 10 HEPES, 11.1 glucose (pH 7.1). Thereafter, hearts were perfused for 30 min with solution A supplemented with 0.2 mg ml^-1^ collagenase (type D, Roche Molecular Biochemicals) and the Ca^2+^ concentration was gradually increased during digestion. After enzymatic digestion, the hearts were perfused for 10 min with solution A and ventricles were cut into several pieces before myocytes were separated by filtration through a nylon mesh.

### Stimulation of Cardiomyocytes

To avoid the interference from endogenous adenosine, adenosine deaminase (5 units/ml) was present under all experimental conditions. In order to get an insight into the signaling via A_2A_-AR we used the following highly selective antagonists for pre-incubation: 1 μM DPCPX (A_1_-AR antagonist) or 1 μM ZM 241385 (A_2A_-AR antagonist). For subsequent stimulation activity 1 μM CGS 21680 [A_2A_-AR agonist (Boknik et al., 2009)] or isoproterenol was added for 10 min at 37°C. After denaturation with 0.1 M HCl at 95°C and centrifugation the supernatant was stored at -20°C for cAMP quantification and the pellet was dissolved in 0.1 M NaOH for subsequent determination of the protein concentration by the assay according to Bradford. For determination of the phosphorylation state the incubation was terminated by adding SDS solution according to Laemmli and samples were subjected SDS PAGE and Western Blotting.

### Contractile Function

Mice were anesthetized by i.p. injection of pentobarbital sodium (50 mg kg^-1^) and hearts were excised. Right and left atria were dissected from isolated A_2A_-AR transgenic and wild type (WT) mice hearts and mounted in an organ bath. Left atrial preparations were continuously electrically stimulated (field stimulation) with each impulse consisting of 1 Hz, with a voltage of 10–15% above threshold and 5 ms duration. Right atrial preparations were allowed to contract spontaneously. The bathing solution contained (in mM) NaCl 119.8, KCl 5.4, CaCl_2_ 1.8, MgCl_2_ 1.05, NaH_2_PO_4_ 0.42, NaHCO_3_ 22.6, Na_2_EDTA 0.05, ascorbic acid 0.28 and glucose 5.0, continuously gassed with 95% O_2_ and 5% CO_2_ and maintained at 35°C resulting in a pH of 7.4. Signals detected via an isometric force transducer were amplified and continuously recorded. CGS 21680, ZM 241385or isoproterenol (1 μM each) were cumulatively applied with approximately 20 min for each compound. Contraction experiments were performed after addition of adenosine deaminase (1 μg ml^-1^) and DPCPX (1 μM) to avoid interference from endogenous adenosine or A_1_ adenosine receptor activation.

### Langendorff-Perfused Hearts

Heart preparations were utilized as described previously (Kirchhefer et al., 2014). Mice were anesthetized intraperitoneally with 2.0 g kg^-1^ body weight urethane and treated with 1.5 units of heparin. The hearts were removed from the opened chest, immediately attached by the aorta to a 20-gauge cannula, and perfused retrogradely under constant pressure of 50 mmHg with oxygenized Krebs-Henseleit buffer (37.4°C) containing 118 mM NaCl, 25 mM NaHCO_3_, 0.5 mM Na-EDTA, 4.7 mM KCl, 1.2 mM KH_2_PO_4_, 1.2 mM MgSO_4_, 2.5 mM CaCl_2_, and 11 mM glucose in an isolated heart system (Hugo Sachs Elektronik, March-Hugstetten, Germany). The heart preparations were allowed to equilibrate for 30 min before measurements. Hearts were stimulated at 8 Hz and heart rate, aortic pressure, and LV pressure were measured and monitored continuously. The first derivative of LV pressure (+dP/dt and -dP/dt) was calculated (ISOHEART Software, Hugo Sachs Elektronik). To generate global ischemia, the perfusion was stopped for 20 min (or 40 and 60 min, respectively) and thereafter the hearts were reperfused for 40 min.

### Quantification of cAMP

Measurements of intracellular cAMP levels were performed by using the Biotrak direct enzyme immunoassay (GE Healthcare, Amersham, Chalfont St. Giles, United Kingdom) according to the manufacturer’s instructions. The cAMP containing supernatant was acetylated (1:40 with acetylation reagent, 1 volume acetic anhydride + 2 volume triethylamine), and divided into the donkey anti-rabbit IgG coated wells of a microtiter plate prefilled with anti-cAMP serum. Competition between unlabeled cAMP in the sample and a fixed quantity of peroxidase-labeled cAMP, for a limited number of binding sites on the cAMP specific antibody allowed quantification of intracellular cAMP.

### Radioligand Binding Experiments

Freshly obtained ventricular tissue was manually minced in small slices. Homogenization was performed at 4°C with Polytron (PT-MR 3000, Kinematica, Lucerne, Switzerland) three times for 30 s at the speed of 20,000 rpm in buffer A, containing sucrose 250 mM, histidine 30 mM (pH = 7.4) and thereafter using Virsonic 100 (VirTis, Gardiner, NY, United States) for 2 s. This suspension was centrifuged for 10 min at 1,500 × *g* and 4°C (Varifuge 3.0R, Heraeus, Hanau, Germany). The supernatant was centrifuged at 45,000 × *g* (Beckman Avanti J-20XP, Beckman Coulter, Palo Alto, CA, United States). The resulting supernatant was stored at -20°C. The pellet was resuspended in buffer B, containing KCl 600 mM, histidine 30 nM (pH = 7.0) and again centrifuged for 45 min at 64,000 × *g*. The pellet was resuspended in 250 μl buffer C, containing sucrose 250 mM, histidine 10 mM (pH = 7.0) and stored at -80°C until it was used for ligand binding experiments.

The density of A_2A_-AR was determined using [^3^H]-CGS 21680. Membrane proteins were diluted to the concentration of 2 μg μl^-1^. The binding assays contained 40 μg of membranes in A_2A_-TG and 400 μg in WT and in addition 8 nmol of [^3^H]-CGS 21680 in a final volume of 200 μl (A_2A_-TG) and 2 ml (WT), which contained also 10 U ml^-1^ adenosine deaminase. Unspecific binding was determined by addition of 1 μM ZM 241385. Binding assay was performed by incubation at 37°C for 90 min. Initially, the experiments for time dependency of ligand association were performed for 15–120 min. Furthermore, protein linearity was measured in the range of 20–120 μg protein. In order to determine B_max_ and K_D_ 16 increasing concentrations of [^3^H]-CGS 21680 from 0.1 to 20 nM were used. For assessment of the specificity of radioligand binding incubations in the presence of the A_1_-AR antagonist PSB36 (100 nM) as well as of the A_3_-AR antagonist MRS1220 were performed. Incubations were terminated by filtration (filter type GF/52, Schleicher und Schuell, Dassel, Germany). Radioactivity bound to the filters was determined by adding 5 ml scintillation fluid (Ultima Gold, Perkin Elmer, Rodgau, Germany) in a counter (Canberra Packard 1600TR, Dreieich, Germany).

### Data Analysis

Data shown are means ± SE. Statistical significance was estimated by analysis of variance followed by Bonferroni’s *t*-test or using the χ^2^-test as appropriate. A *p*-value < 0.05 was considered as significant.

### Drugs and Materials

All other chemicals were of analytical grade. Deionized water was used throughout the experiments. Stock solutions were freshly prepared daily.

## Results

### A_2A_-AR Overexpression

**FIGURE 1:**
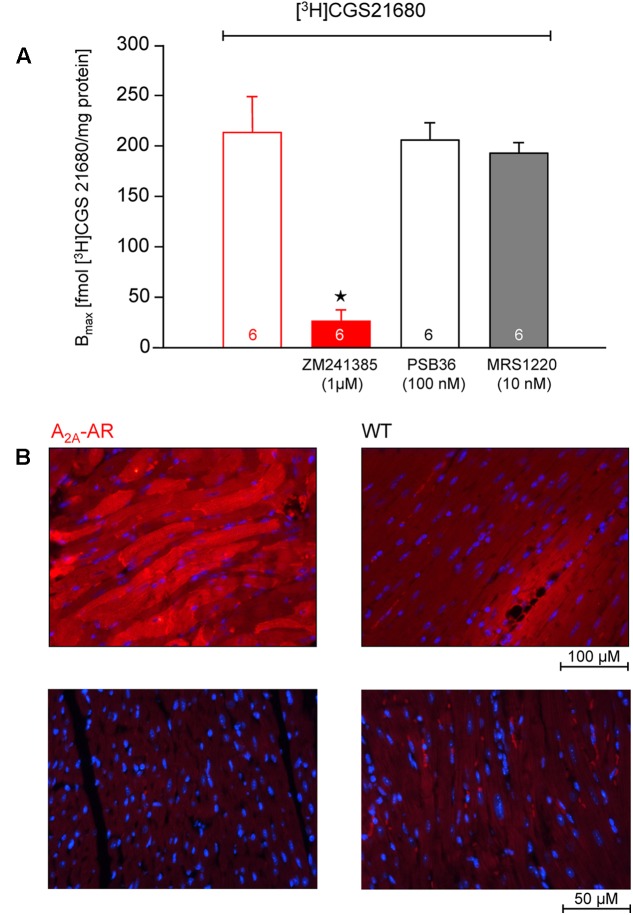
**(A)** Radioligand binding studies in membrane fraction of A_2A_-AR-overexpressing animals. The experiments were performed as described in Section “Materials and Methods.” The binding of [^3^H]-labeled A_2A_-AR-agonist CGS 21680 could be almost completely blocked by addition of A_2A_-AR-antagonist ZM 241385, whereas A_1_-AR-antagonist PSB36 and A_3_-AR-antagonist MRS 1220 did not affect the binding of [^3^H]-CGS 21680. No specific binding was detectable in membrane fraction from WT animals. **(B)** Immunohistological staining of ventricular tissue from A_2A_-AR-overexpressing and wild type (WT) animals. A_2A_-AR was visualized using a monoclonal antibody against A_2A_-AR and goat anti-mouse antibody conjugated to Alexa Fluor 594 (red, upper row). The nuclei were counterstained using DAPI (blue). Note staining of cardiomyocytes from A_2A_-AR animals, mainly in the vicinity of the sarcolemma. Lower row: negative controls with omission of the primary antibody. ^⋆^*p* < 0.05 vs. [^3^H]-CGS 21680 alone.

In transgenic mice which overexpress the A_2A_-AR, we noted that the A_2A_-AR could be detected on the protein level for the following reasons. First of all, using very sensitive radioligand binding experiments, we could detect binding of the [^3^H]-labeled A_2A_-AR-agonist CGS 21680 samples form A_2A_-TG hearts. This binding of [^3^H]-labeled A_2A_-AR-agonist CGS 21680 could be almost completely blocked by addition of the (non-radioactively labeled) A_2A_-AR-antagonist ZM 241385, whereas (non-radioactively labeled) A_1_-AR-antagonist PSB36 or (non-radioactively labeled) A_3_-AR-antagonist MRS 1220 did not affect the binding of [^3^H]-CGS 21680. Interestingly, under the same experimental conditions used in samples from A_2A_-TG heart, no specific binding of A_2A_-AR using the [^3^H]-labeled A_2A_-AR-agonist CGS 21680 could be established in samples of the hearts from WT littermates (**Figure 1A**). Secondly, the A_2A_-AR could be visualized using immunohistology in A_2A_-TG hearts. We tentatively identified the localization of the receptor in A_2A_-TG to the vicinity of the sarcolemma (**Figure 1B**). Relative heart weight, an established parameter of cardiac hypertrophy, did not differ in whole hearts, ventricles or right atria between A_2A_-TG and WT (**Table 1**). These data exclude a cardiac hypertrophy of A_2A_-AR hearts at the two time points studied.

**Table 1:** Relative weight [with regard to body weight (BW)] of whole hearts (HW/BW), ventricles (V/BW), right atria (RA/BW) and left atria (LA/BW) in wild type (WT) and A_2A_-AR overexpressing animals at the age of 12 and 30 weeks.

	12 weeks		30 weeks	
		
	WT (*n* = 65)	A_2A_-AR (*n* = 51)	WT (*n* = 32)	A_2A_-AR (*n* = 36)
HW/BW (mg/g)	4.79 ± 0.06	4.73 ± 0.06	4.88 ± 0.07	4.89 ± 0.11
V/BW (mg/g)	4.08 ± 0.04	4.03 ± 0.06	4.05 ± 0.05	4.11 ± 0.02
RA/BW (mg/g)	0.37 ± 0.01	0.38 ± 0.01	0.38 ± 0.01	0.41 ± 0.02
LA/BW (mg/g)	0.18 ± 0.01	0.15 ± 0.02	0.17 ± 0.01	0.17 ± 0.01


*No significant differences were noted between WT and A_*2A*_-AR overexpressing animals.*

### Expression of Sarcoplasmic and G Proteins

The expression of CSQ (calsequestrin), SERCA [sarcoplasmic reticulum (SR) Ca^2+^ ATPase 2a], and triadin and junctin (both proteins located to the junctional SR), were unchanged between WT and A_2A_-TG. However, PLB (phospholamban) expression was tentatively but not significantly increased in A_2A_-TG and is currently regarded as unchanged. Interestingly, at weeks 12 and 30 the expression of Gsα was lower in A_2A_-TG compared to WT ventricles (**Table 2**). On the opposite note, in ventricle from 30 weeks of A_2A_-TG mice an increase of Giα proteins was detected, but not at age 12 weeks or in atrial preparations (**Table 2**).

**Table 2:** Expression of cardiac regulatory proteins in ventricles of wild type (WT) and A_2A_-AR overexpressing animals at the age of 12 and 30 weeks.

	12 weeks		30 weeks	
		
	WT	A_2A_-AR	WT	A_2A_-AR
CSQ	100 ± 11	100 ± 11	100 ± 7	89 ± 5
Gsα	100 ± 6	64 ± 4^∗^	100 ± 7	66 ± 6^∗^
Giα	100 ± 11	109 ± 10	100 ± 10	148 ± 13^∗^
JCN	100 ± 15	99 ± 15	100 ± 9	109 ± 11
PLB	100 ± 15	148 ± 23	100 ± 11	140 ± 16
SERCA	100 ± 16	119 ± 20	100 ± 10	125 ± 11
TRD	100 ± 16	100 ± 15	100 ± 6	120 ± 19


*Data are given as % of expression in corresponding WT animals (*n* = 6 each). CSQ, calsequestrin; Gsα, α-subunit of stimulatory G protein; Giα, α-subunit of inhibitory G protein; JCN, junctin; PLB, phospholamban; SERCA, sarcoendoplasmatic Ca^*2*+^-ATP-ase; TRD, triadin. ^∗^*p* < 0.05 vs. corresponding WT.*

### Receptor Signaling

**FIGURE 2:**
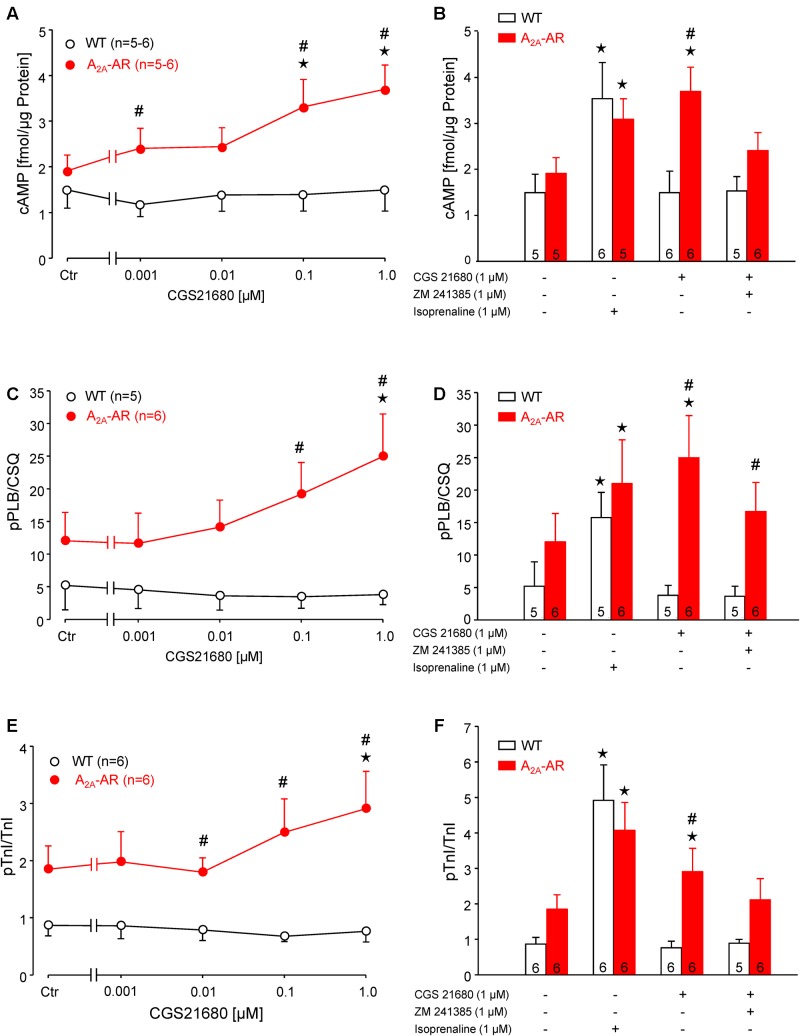
**(A,B)** cAMP content in isolated ventricular cardiomyocytes from WT and A_2A_-AR-overexpressing animals. cAMP content was determined by Biotrak direct Enzymimmunoassay. Note concentration dependent increase of cAMP content by A_2A_-AR agonist CGS 21680 in A_2A_-AR cardiomyocytes **(A)**; this effect could be completely blocked by A_2A_-AR antagonist ZM 241385. The effect of β-adrenoceptor agonist isoproterenol on cAMP content did not differ between WT and A_2A_-AR **(B)**. **(C,D)** Phosphorylation of phospholamban at serine-16 (pPLB). **(E,F)** Phosphorylation of the inhibitory subunit of troponin (pTnI). The phosphorylation state of PLB and TnI was assessed by phosphorylation specific antibodies. The signals obtained using phosphorylation specific antibodies were normalized to the corresponding protein expression. Note concentration dependent increase of pPLB **(C)** and pTnI **(E)** by A_2A_-AR agonist CGS 21680 in A_2A_-AR cardiomyocytes; these effects were abolished by A_2A_-AR antagonist ZM 241385. The effects of isoproterenol on protein phosphorylation were comparable in WT and A_2A_-AR **(D,F)**. ^⋆^*p* < 0.05 vs. Ctr; ^#^*p* < 0.05 vs. WT.

The A_2A_-AR agonist CGS 21680 concentration-dependently increased the cAMP content in ventricular cardiomyocytes from A_2A_-TG but not in WT cardiomyocytes (**Figure 2A**). The increase in cAMP content in the presence of 1 μM CGS 21680 under these conditions was similar to the response of a maximum effective concentration (1 μM) of the β-adrenoceptor agonist isoproterenol (**Figure 2B**). These findings are consistent with the fact that we measured a concentration dependent effect of CGS 21680 on phospholamban phosphorylation in isolated ventricular cardiomyocytes from A_2A_-TG but not WT hearts (**Figure 2C**). We used in these experiments adenosine deaminase (ADA) that is an enzyme that rapidly degrades adenosine to inosine. Inosine is not an agonist at adenosine receptors. The inclusion of ADA was, in our hands, crucial in order to detect changes in protein phosphorylation (see **Figure 2** in Gupta et al., 1993a). Importantly, we also noted a similar increase in isoproterenol-induced phospholamban phosphorylation in cardiomyocytes from A_2A_-TG and WT (**Figure 2D**). Moreover, it is noteworthy that the maximum increase in phospholamban phosphorylation was similar in WT in the presence of isoproterenol vs. CGS 21680 in A_2A_-TG cardiomyocytes (**Figure 2D**). In addition, we also noted a similar increase in CGS 21680-induced phosphorylation of troponin I in ventricular cardiomyocytes from A_2A_-TG hearts but not from WT hearts (**Figure 2E**). Likewise, it is notable that isoproterenol increased the phosphorylation of troponin I not only in A_2A_-TG cardiomyocytes but also WT cardiomyocytes (**Figure 2F**). Furthermore, the maximum increase in TnI phosphorylation was similar in WT in the presence of isoproterenol vs. CGS 21680 in A_2A_-TG myocytes (**Figure 2F**). Moreover, the increase of cAMP-content and protein phosphorylation in A_2A_-TG in the presence of the A_2A_-AR agonist CGS 21680 could be attenuated by the additional presence of the A_2A_-AR antagonist ZM 241385 (**Figure 2**, bar diagrams).

### Contractility

**FIGURE 3:**
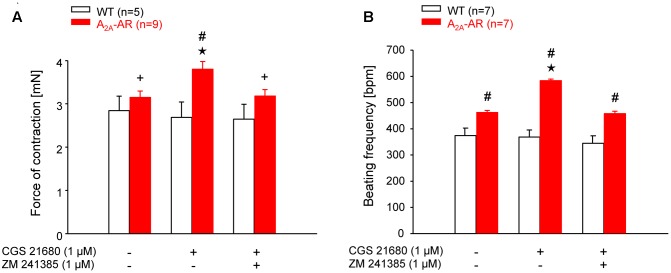
**(A)** Force of contraction (FOC) in isolated electrically driven left atria. **(B)** Beating frequency (BF) in isolated spontaneously beating right atria. The contraction experiments were performed in the presence of A_1_-AR antagonist DPCPX (1 μM). Note the higher basal BF and the increase of FOC and BF by A_2A_-AR agonist CGS 21680 in atria of A_2A_-AR overexpressing animals. These effects were abolished in the presence of A_2A_-AR antagonist ZM 241385. ^⋆^*p* < 0.05 vs. Ctr; ^#^*p* < 0.05 vs. WT; ^+^*p* < 0.05 vs. CGS 21680 alone.

The mean values of developed tension under basal conditions (no exogenous pharmacological stimulation) in electrically driven left atrial preparations (12 and 30 weeks) were higher at both ages in A_2A_-TG than WT but did not gain statistical significance (**Table 3**). However, basal beating rate in A_2A_-TG was higher than in WT (30 weeks **Table 3**) Contractile studies in the organ bath were performed in the additional presence of the A_1_-AR antagonist DPCPX (Neumann et al., 1989). This was done in order to exclude possible interference of any residual adenosine released from cells with the A_1_-AR (stimulation of A_1_-AR in the atrium exerts negative inotropic and negative chronotropic effects [mouse (Boknik et al., 1997)]. At weeks 12 and 30, the A_2A_-AR agonist CGS 21680 (1 μM) increased force of contraction in electrically stimulated A_2A_-TG left atrial preparations but not in electrically stimulated WT atrial preparations (**Figure 3A**). In electrically stimulated A_2A_-TG left atrial preparations, the positive inotropic effects of CGS 21680 (1 μM) were attenuated by the A_2A_-AR antagonist ZM 241385 (1 μM, **Figure 3A**). Similarly, 1 μM CGS 21680 was able to increase the beating rate at 12 and 30 weeks in spontaneously beating A_2A_-TG right atrial preparations but not in spontaneously beating WT right atrial preparations (**Figure 3B**). This increase in beating rate in spontaneously beating A_2A_-TG right atrial preparations was attenuated by the A_2A_-AR antagonist ZM 241385 (1 μM, **Figure 3B**).

**Table 3:** Basal force of contraction (FOC) in electrically driven left atria and basal beating rate (BR) in spontaneously beating right atria at the age of 12 and 30 weeks in wild type (WT) and A_2A_-AR overexpressing animals.

	12 weeks		30 weeks	
		
	WT	A_2A_-AR	WT	A_2A_-AR
FOC (mN)	2.49 ± 0.25 (19)	2.91 ± 0.25 (23)	3.23 ± 0.23 (19)	3.69 ± 0.20^#^ (25)
BR (bpm)	441 ± 12 (12)	469 ± 22 (8)	410 ± 27 (11)	496 ± 16^∗^ (10)


*The measurements were performed after preincubation with adenosine deaminase (30 min, 1 μg/ml) to avoid interference from endogenous adenosine. Numbers of atria are given in parentheses. ^∗^*p* < 0.05 vs. corresponding WT; ^#^*p* < 0.05 vs. A_*2A*_-AR at the age of 12 weeks.*

### β-Adrenergic Stimulation

**FIGURE 4:**
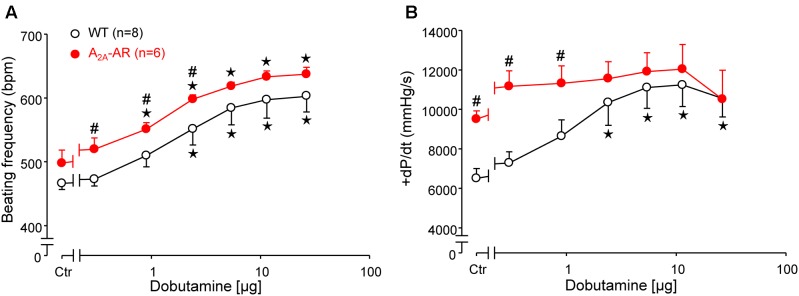
Hemodynamic parameters assessed by left ventricular catheterization in anesthetized WT and A_2A_-AR overexpressing animals as described in Section “Materials and Methods.” Effects of dobutamine on beating frequency (BF, **A**) and maximum rate of left ventricular pressure development (+dP/dt, **B**). Note the higher basal +dP/dt and the lack of dobutamine effect on +dP/dt in A_2A_-AR overexpressing animals. ^⋆^*p* < 0.05 vs. Ctr; ^#^*p* < 0.05 vs. WT.

**FIGURE 5:**
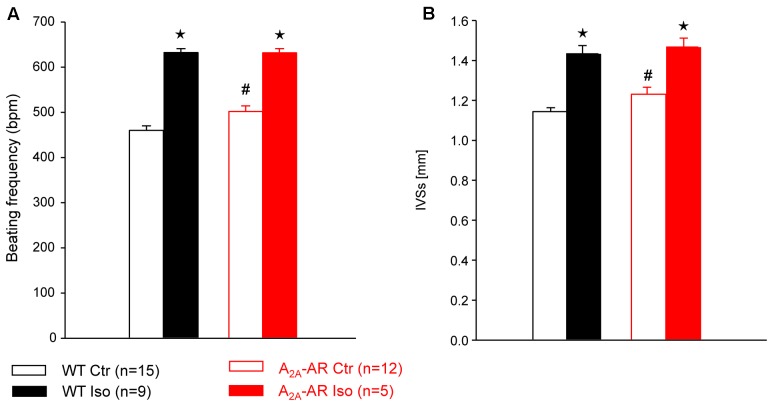
Hemodynamic parameters assessed by Doppler echocardiography in anesthetized WT and A_2A_-AR overexpressing animals. Note the increased basal beating frequency **(A)** and interventricular systolic septum thickness (IVSs, **B**) in A_2A_-AR overexpressing animals. The effects of β-adrenoceptor agonist isoproterenol on both parameters were comparable between WT and A_2A_-AR overexpressing animals. ^⋆^*p* < 0.05 vs. Ctr; ^#^*p* < 0.05 vs. WT.

In A_2A_-TG and WT, invasively left ventricular function was assessed by a catheter while drug was continuously infused with a syringe pump into the jugular vein. The increase of the beating rate to infusion of dobutamine (a clinically used β-adrenoceptor agonist) was similar in A_2A_-TG and WT (**Figure 4A**). Basal left +dP/dT was higher in A_2A_-TG compared to WT animals (**Figure 4B**). However, in WT, dobutamine increased +dP/dT to a higher extent than in A_2A_-TG (**Figure 4B**). In subsequent echocardiographic studies, basal heart rate and interventricular systolic septum thickness were increased in A_2A_-TG compared to WT. However, the effects of β-adrenoceptor agonist isoproterenol on both parameters were comparable between WT and A_2A_-TG animals (**Figure 5**).

### Ischemia/Reperfusion Experiments

**FIGURE 6:**
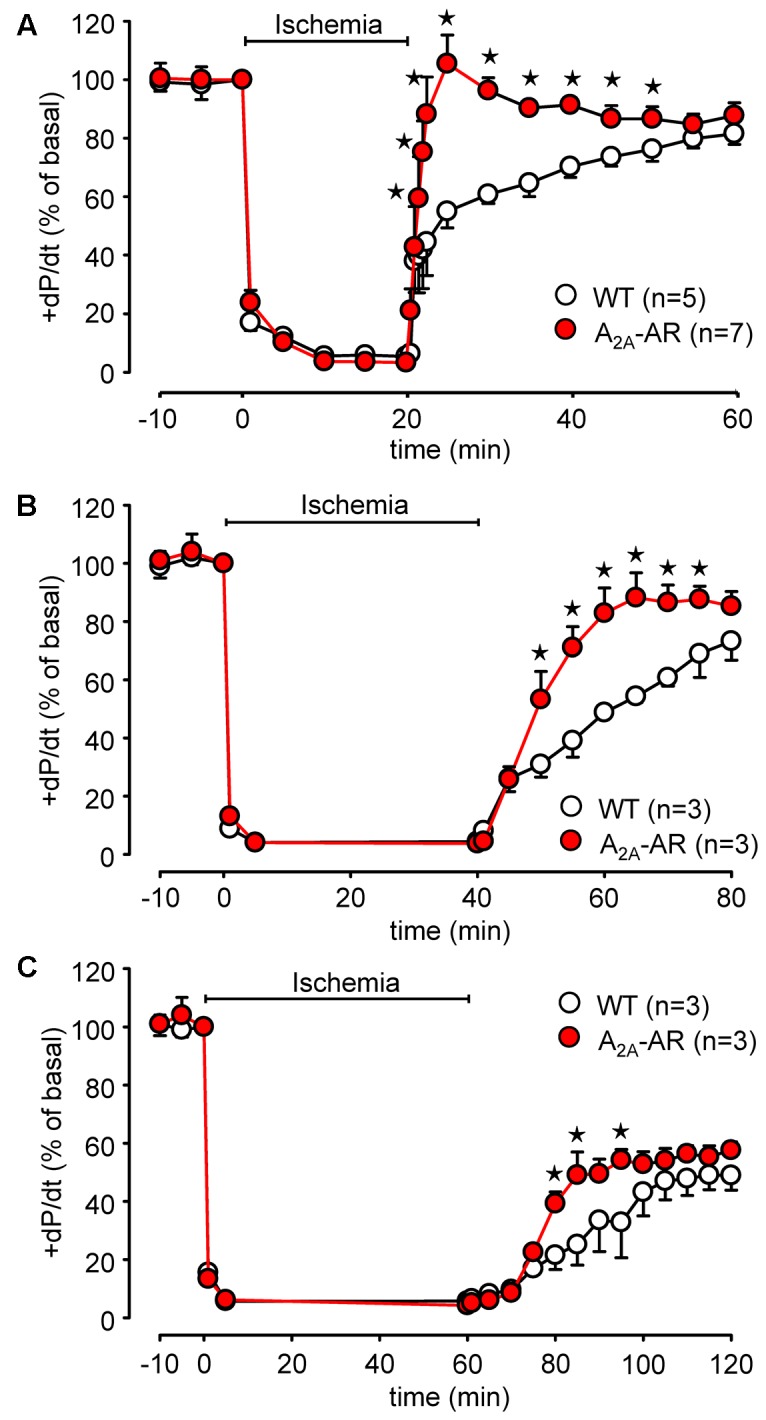
Comparison of 20 min **(A)**, 40 min **(B)**, and 60 min **(C)** of global ischemia in isolated Langendorff-perfused hearts from WT and A_2A_-AR overexpressing animals. Maximum rate of left ventricular pressure development (+dP/dt) is presented as % of basal value. ^⋆^*p* < 0.05 vs. WT.

**FIGURE 7:**
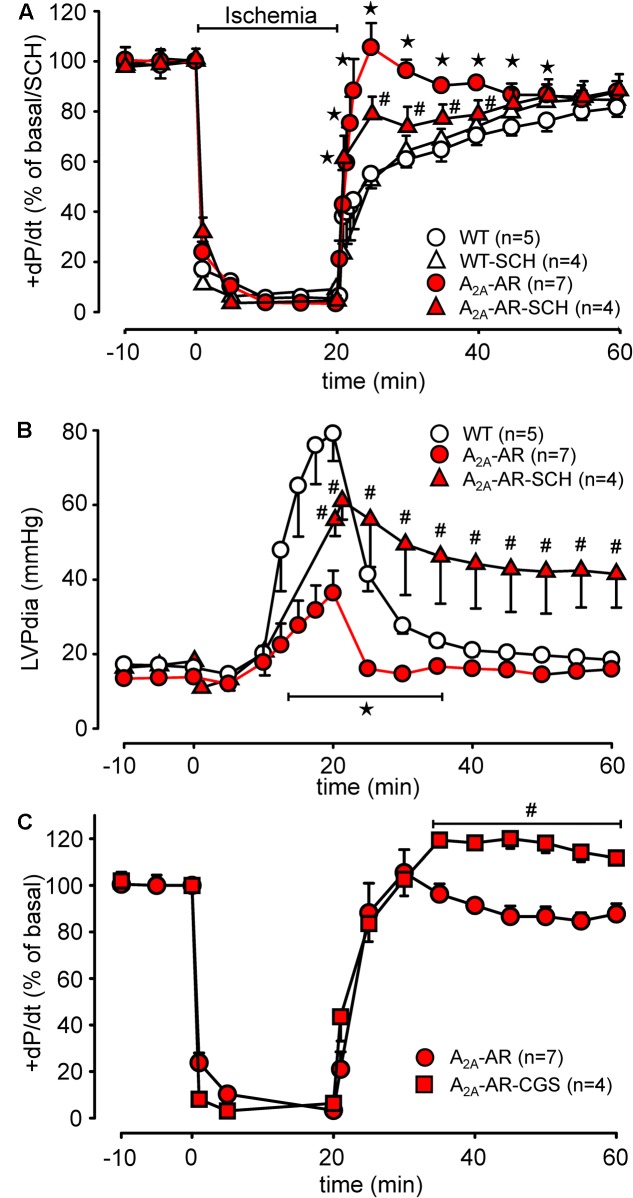
Hemodynamic parameters during global ischemia with subsequent reperfusion assessed in isolated Langendorff-perfused hearts from WT and A_2A_-AR overexpressing animals. **(A)** Maximum rate of left ventricular pressure development (+dP/dt) as % of basal value with and without the A_2A_-AR antagonist SCH 442416 (SCH, 100 nM). **(B)** Left ventricular end diastolic pressure with and without the A_2A_-AR antagonist SCH 442416 (SCH, 100 nM). **(C)** Effect of A_2A_-AR agonist CGS 21680 (1 μM) administered during reperfusion on +dP/dt in A_2A_-AR overexpressing hearts. Note the higher basal +dP/dt, markedly prolonged time to ischemic contracture as well as the additional effect of A_2A_-AR agonist CGS 21680 on the +dP/dt in A_2A_-AR overexpressing hearts during reperfusion. ^⋆^*p* < 0.05 vs. WT; ^#^*p* < 0.05 vs. A_2A_-AR.

Global ischemia was induced in paced (8 Hz) isolated Langendorff-perfused hearts by complete stop of the perfusion. Hearts were paced because differences in heart rate, alone, might influence contractility. Basal heart rates before pacing amounted to 421.5 ± 14.5 bpm for WT and 467.8 ± 11.7 bpm for TG (*p* < 0.05 vs. WT, *n* = 5–7). During global ischemia (20 min duration), contractile response (here +dP/dt) rapidly stopped (**Figure 6A**). Protective effects were smaller but also present after 40 and 60 min of global ischemia and reperfusion (**Figures 6B,C**). Notably, basal rates of pressure development were higher in A_2A_-TG than in WT (6,097.3 ± 213.9 mmHg/s vs. 3,657.7 ± 144.7 mmHg/s, *p* < 0.05 vs. WT, *n* = 5–7). Upon reperfusion, cardiac contractile response gradually resumed and relative similar pressure values were reached as before the ischemia in A_2A_-TG and WT (**Figure 7A**). It is noteworthy that diastolic function is quite sensitive to ischemia in A_2A_-TG (**Figure 7B**) and the increase in diastolic pressure and its normalization as well as increase and normalization of rates of pressure development were sensitive to 100 nM SCH 442416, an A_2A_-AR antagonist (**Figures 7A,B**). Moreover, CGS 21680 was able to hasten and elevate the percentile restoration of ventricular + dP/dT (**Figure 7C**).

## Discussion

In case of β-adrenergic stimulation and hypoxia, the amount of extracellular adenosine increases in the coronary system (Bardenheuer et al., 1987). Adenosine concentrations are much higher in cardiomyocytes compared to in the extracellular space. Thus, there is a huge concentration gradient for adenosine across the cell membrane and if for example during a myocardial infarction a necrosis occurs, a large amount of adenosine can be released from dying cardiomyocytes.

Adenosine can be a degradation product of ATP but it can also be produced *de novo*. Finally, adenosine deaminases are responsible for metabolizing and thus inactivating adenosine. Adenosine acts via adenosine receptors. All adenosine receptors are a subgroup of *P*-purinoceptors which are separated further into P_1_ - or P_2_ -purinoceptors (Shryock and Belardinelli, 1997; Burnstock, 2017). P_1_-purinoceptors are more sensitive to adenosine than to ATP, the opposite holds true for P_2_-purinoceptors (Shryock and Belardinelli, 1997; Burnstock, 2017).

We succeeded in expressing exogenous A_2A_-AR in cardiomyocytes: importantly we can only detect radioligand binding to the A_2A_-AR in the A_2A_-TG heart but not in WT heart. None of the biochemical or physiological parameters studied in an integrated and fairly complete approach supports the functional presence of A_2A_-AR in WT myocytes, e.g., A_2A_-AR agonists did not increase cAMP, protein phosphorylation, beating rate or force of contraction in cardiomyocytes or isolated cardiac preparation from WT mouse hearts. In contrast, a selective A_2A_-AR agonist CGS 21680 increased cAMP content, protein phosphorylation and contractility in A_2A_-TG preparations.

In genetically modified hearts, homeostatic changes in the expression of other genes occur (Engelhardt et al., 2001; Lankford et al., 2002). Similar homoeostatic gene changes are plausible in the present transgenic model. Hence, we interpret the decrease in Gs as a protection against persistent basal activity of the A_2A_-AR leading to chronic elevation of AC activity and cAMP levels. Likewise, it is tempting to assume that the increased Gi levels in order to decrease cAMP levels to normal levels. The unchanged expression of main Ca^2+^ handling proteins of the SR facilitates the interpretation of our contractile data and may be seen in light of our previous work where we studied the function of for example triadin or junctin overexpressing mice (Kirchhefer et al., 2001, 2003, 2004a,b, 2006, 2007; Buchwalow et al., 2004; Kirchhof et al., 2007).

There are a number of studies *in vitro* and *in vivo* showing the overexpression of a Gs coupled receptor *per se* can increase the coupling of receptor and cAMP accumulation. This can result in increased basal effects of the generated cAMP and manifests itself in an increase in basal cAMP and/or increase in PLB phosphorylation, increased basal contraction in left atrial preparations (or intact catheterized hearts of on echocardiography) and/or increased beating rate in isolated right atrial preparation (or in isolated perfused hearts or catheterized hearts of echocardiography). We reported this chain of events before for 5HT_4_ receptor overexpressing mice (Gergs et al., 2013). In part these alterations are present in this mouse model like an increase in basal beating rate (isolated atrial preparations, catheterized hearts, echocardiography).

The continuous stimulation of cAMP content by the overexpressed A_2A_-AR might explain why higher beating rates are noted in 30 week old A_2A_-TG right atrial preparations. It is possible that the compensatory mechanisms detected and discussed above lose their effectiveness at this age (but note that they are apparently sufficient to keep parameters in the range of WT at age 12 weeks).

Our interpretation of a functional A_2A_-AR expression (or overexpression, depending on data interpretation) is supported by the fact that an A_2A_-AR agonist increases force in left atrial preparations and beating rate in isolated right atrial preparations. Consistent with this the inotropic and chronotropic effects could be reduced by an appropriate antagonist.

Others have shown that A_1_-AR overexpression as well as A_3_-AR receptor overexpression can protect cardiac contractility against short term ischemia [preconditioning (Matherne et al., 1997; Black et al., 2002)].

Others have reported before on a constitutive (Chan et al., 2008) or inducible overexpression (Hamad et al., 2010) of the human A_2A_-receptor in the mouse heart. Our data confirm but also extend this previous work: like us they noted an increase in the heart rate in TG.

In contrast to our data they noted cardiac hypertrophy (**Table 1**; Chan et al., 2008). Similar to Chan et al. (2008) we detected histologically A_2A_-AR overexpression in myocytes, increased expression of A_2A_-AR in Western Blots, and increased levels of Giα and increased basal contractility (in invasive left ventricular measurements prior to any drug application). We extend those data (besides that we used a similar but not identical promoter to drive cardiomyocyte specific overexpression) by describing a reduced expression of Gs in TG, studying isolated atrial preparations (elucidation the regional cardiac effects of A_2A_-AR-overexpression) and by studying the signal transduction in more detail. We concur with their data that cAMP (they measured adenylyl cyclase activity) is not increased in TG under basal conditions and therefore basal phosphorylation state of TnI and phospholamban was not enhanced (which they did not measure). We further extend their work by showing that the overexpressed A_2A_-receptor exhibits the expected pharmacology: namely the A_2A_-AR agonist CGS 21680 concentration dependently increased cAMP content in TG (but not WT) and likewise, probably by stimulating the activity of PKA, leads to increased phosphorylation state of phospholamban on its PKA sensitive phosphorylation site and in increased phosphorylation state addition of TnI.

While the group of Feldman elegantly used their model to study *in vivo* functional interaction of A_1A_ coexpression with A_2A_-receptors (Chan et al., 2008), the protective role of A_2A_ in pressure overload (aortic banding: Hamad et al., 2012) or protection against the cardiodepressant role of chronic Adriamycin treatment (Hamad et al., 2010), we used our model to initiate a first study on the putative protective role of A_2A_-overexpression in reperfusion of the heart, which to the best of our knowledge has not been studied before, while a protective role of A_2A_-AR in other species using pharmacological stimulation has been described. We would argue that overexpression of the receptor adds substantially on our mechanistically knowledge in reperfusion injury because, pharmacological stimulation will not allow delineating which cells are involved in a putative protective effect of A_2A_-receptors: they are expressed (at least functionally) not only in cardiomyocytes but also in cardiac endothelial cells and smooth muscle cells. Hence, any effect we observe in perfused hearts (in the absence of drug application) is probably due to the action of endogenously produced adenosine on the human A_2A_-AR in cardiomyocytes. Another advantage of our approach (compared to other reports, e.g., Jordan et al., 1997) is that we used isolated buffer perfused hence A_2A_-receptors in the blood cannot confound our data (which can occur, see, Yang et al., 2005). Others have failed to see any effect of A_2A_-KO vs. WT in isolated perfused heart on reperfusion injury (Zhan et al., 2011, review: McIntosh and Lasley, 2012). Based on our data, we would argue that it might be due to the loss of A_2A_ not only in cardiomyocytes (which we would predict to be deleterious) but also on endothelial, smooth muscle and fibroblasts (for review cell specific expression of A_2A_-AR, e.g., McIntosh and Lasley, 2012). It would be interesting to generate and study reperfusion in mice with cardiomyocytes specific A_2A_-AR KO.

Finally, the data on ischemia may suggest that the A_2A_-AR may play a protective role of this receptor in ischemia. It might be speculated that alterations of this receptor occur physiologically in myocardial ischemia (for instance during infarction and stenting of the vessel). These receptors may therefore be a target for pharmacological treatment of reperfusion injury. However, more detailed studies are called for and are expected with interest.

Moreover, one can ask whether this model has (patho)-physiological relevance. In this regard it might be of interest that during ischemia in isolated perfused hearts the expression of A_2A_-AR increases (Morrison et al., 2006). A_2A_ agonists like regadenoson are clinically used to detect latent ischemia in patients (Cerqueira et al., 2008).

### Study Limitations

Furthermore, the direct comparison with the human situation is not easy. In earlier work, we reported that adenosine can in some atrial samples from patients exert a positive inotropic effect (Gergs et al., 2009). However, the effect was A_1_ receptor mediated. We have not been able to perform similar experiments in human ventricular tissue (for lack of material at our institutions). However, some years ago we reported at least an increase in the mRNA of A_2A_-AR in failing human ventricular samples compared to non-failing (Stein et al., 1998). Thus, it is tempting to speculate that A_2A_-AR may sustain contractility in end stage human heart failure. Hence, A_2A_-AR may serve a physiological role in humans and, as shown for adenoviral expression of adenylate cyclase, genetic manipulation of A_2A_-AR may offer a novel venue for treating human heart failure.

## Conclusion

We describe a transgenic mouse with A_2A_-AR overexpression to cardiomyocytes using the established alpha myosin heavy chain promoter. Moreover, transgenic A_2A_-AR is functional after drug-induced stimulation to increase force and beating rate in mouse hearts or mouse heart preparations and the transgenic A_2A_-AR can protect the mouse heart against ischemia.

## Author Contributions

KD, JE, SG-W, LF, FS, and UK performed the research. PB, FM, WS, and JN designed the research study. PB, PK, NZ, UK, and JN analyzed the data. PB, UG, and JN wrote the paper.

## Conflict of Interest Statement

The authors declare that the research was conducted in the absence of any commercial or financial relationships that could be construed as a potential conflict of interest.
